# Supplementary material for: Co-aggregation and secondary nucleation in the life cycle of human prolactin/galanin functional amyloids
Source: eLife. 2022 Mar 8;11:e73835. doi: 10.7554/eLife.73835 (PMC8993219; doi:10.7554/eLife.73835)
Supplement: Figure 1—source data 2. [file elife-73835-fig1-data2.docx]

Figure 1-table supplement 1: Protein/peptide hormone sequences used in the study

| Protein | Sequence  50  10 |
| --- | --- |
| Human prolactin (PRL) | 30  40  20  1 LPICPGGAAR CQVTLRDLFD RAVVLSHYIH NLSSEMFSEF DKRYTHGRGF  100  90  80  70  60    51 ITKAINSCHT SSLATPEDKE QAQQMNQKDF LSLIVSILRS WNEPLYHLVT  150  140  130  120  110  101 EVRGMQEAPE AILSKAVEIE EQTKRLLEGM ELIVSQVHPE TKENEIYPVW  190  180  170  160  151 SGLPSLQMAD EESRLSAYYN LLHCLRRDSH KIDNYLKLLK CRIIHNNNC  30  10  20 |
| Human galanin (GAL) | 1 GWTLNSAGYL LGPHAVGNHR SFSDKNGLTS  50  10 |
| Human growth hormone (GH) | 40  30  20  1 FPTIPLSRLF DNAMLRAHRL HQLAFDTYQE FEEAYIPKEQ KYSFLQNPQT  100  80  70  60    90  51 SLCFSESIPT PSNREETQQK SNLELLRISL LLIQSWLEPV QFLRSVFANS  150  140  130  120  110    101 LVYGASDSNV YDLLKDLEEG IQTLMGRLED GSPRTGQIFK QTYSKFDTNS  180  170  160    190  151 HNDDALLKNY GLLYCFRKDM DKVETFLRIV QCRSVEGSCG F |
| Human adrenocorticotropic hormone  (ACTH) | 1 SYSMEHFRWG KPVGKKRRPV KVYPNGAEDE SAEAFPLEF  30  20  10 |
