# Supplementary material for: Co-aggregation and secondary nucleation in the life cycle of human prolactin/galanin functional amyloids
Source: eLife. 2022 Mar 8;11:e73835. doi: 10.7554/eLife.73835 (PMC8993219; doi:10.7554/eLife.73835)
Supplement: Figure 4—source data 2. [file elife-73835-fig4-data2.docx]

Figure 4-table supplement 1: Parameter values for surface plasmon resonance spectroscopy (SPR).

| **Protein Combination** | **k_on_ (molar^-1^ second^-1^)** | **K_off_  (second^-1^)** | **K_D_ (μM)** |
| --- | --- | --- | --- |
| PRL-GAL | 9975.66 (± 1138.65) | 0.004 (± 0.002) | 0.41 (± 0.18) |
| PRL-ACTH | 1780.66 (± 533.21) | 0.01 (± 0.001) | 7.24 (± 2.69) |
| GH-GAL | 746.33 (± 112.61) | 0.013 (± 0.002) | 19.71 (± 6.99) |
